# Metabolomics of bronchoalveolar lavage in children with persistent wheezing

**DOI:** 10.1186/s12931-022-02087-6

**Published:** 2022-06-19

**Authors:** Lingfang Liang, Minfei Hu, Yuanling Chen, Lingke Liu, Lei Wu, Chengcheng Hang, Xiaofei Luo, Xuefeng Xu

**Affiliations:** Department of Rheumatology Immunology and Allergy, The Children’s Hospital, Zhejiang University School of Medicine, National Clinical Research Center for Child Health, Hangzhou, 310003 People’s Republic of China

**Keywords:** Airway, Bronchoalveolar lavage, Children, Metabolomics, Persistent wheezing

## Abstract

**Background:**

Recent studies have demonstrated the important role of metabolomics in the pathogenesis of asthma. However, the role of lung metabolomics in childhood persistent wheezing (PW) or wheezing recurrence remains poorly understood.

**Methods:**

In this prospective observational study, we performed a liquid chromatography/mass spectrometry-based metabolomic survey on bronchoalveolar lavage samples collected from 30 children with PW and 30 age-matched infants (control group). A 2-year follow-up study on these PW children was conducted.

**Results:**

Children with PW showed a distinct characterization of respiratory metabolome compared with control group. Children with PW had higher abundances of choline, oleamide, nepetalactam, butyrylcarnitine, l-palmitoylcarnitine, palmitoylethanolamide, and various phosphatidylcholines. The glycerophospholipid metabolism pathway was the most relevant pathway involving in PW pathophysiologic process. Additionally, different gender, prematurity, and systemic corticoids use demonstrated a greater impact in airway metabolite compositions. Furthermore, for PW children with recurrence during the follow-up period, children who were born prematurely had an increased abundance of butyrylcarnitine relative to those who were carried to term.

**Conclusions:**

This study suggests that the alterations of lung metabolites could be associated with the development of wheezing, and this early alteration could also be correlated with wheezing recurrence later in life.

**Supplementary Information:**

The online version contains supplementary material available at 10.1186/s12931-022-02087-6.

## Introduction

Asthma is the most common chronic disease among children with an increased trend of disability-adjusted life-years in the last 30 years [1]. Moreover, asthma frequently begins in early childhood, and in up to half of asthmatics, symptoms commence during childhood. These children usually manifest as repeated wheezing, especially for children younger than two years. Some infants will develop persistent or recurrent wheezing, which is often severe [2]. Additionally, increased evidence indicated that infantile wheezing or persistent wheezing (PW) was strongly associated with the development of asthma later in life [3, 4]. However, the underlying mechanisms between them have not been fully elucidated. Many of biologically plausible mechanisms had suggested the effect of environmental changes early in life on the subsequent development of asthma, including cytokine response, developmental origins of adult disease, and microbial exposure [4–8].

Although the understanding of living organisms at the level of molecular system is still in its infancy, comprehensive investigations of the omics technologies with genomics, transcriptomics, proteomics, and metabolomics will provide a better understanding of the biochemical and biological mechanisms in complex systems [9]. Mass spectrometry (MS)-based metabolomics is at the endpoint of the omics cascade and is the closest to phenotype, mainly offering the systematic analyses of small molecules involving carbohydrates, amino acids, lipids, organic acids and nucleotides generated from the cellular metabolic activity [9, 10]. Metabolomics play an important role in biomarker discovery, predicting response to therapy and potential pathogenic pathways for a variety of complex diseases. As a global profiling strategy, the untargeted metabolomics is used to perform an initial evaluation to shortlist key metabolites with distinct alterations [11].

In addition to acute respiratory distress syndrome, chronic obstructive pulmonary disease and lung tumor [11–13], metabolic alterations of different specimens from asthmatics were confirmed [14–17]. To date, metabolomic studies of childhood asthma have been mainly performed on blood, urine, and exhaled breath condensate samples [10]. Given the core role of airway epithelium in the type 2 immunity and asthma, specimens from the lower respiratory tract can better reflect the pathophysiological process of asthma [18, 19]. A certain respiratory metabolic profile could be associated with PW or recurrence of PW. Here, we performed a liquid chromatography/mass spectrometry (LC/MS)-based metabolomic survey on bronchoalveolar lavage (BAL) samples collected from children with PW, aiming to explore their metabolite compositions. Additionally, we also conducted a follow-up study on these PW patients, observing whether the recurrence of wheezing is related to the specific respiratory metabolomic spectrum.

## Methods

### Study subjects

In this prospective observational study, children with persistent wheezing (PW) and age-matched control children were enrolled. Diagnosis of PW was based on persistent episodes of infantile wheezing [2]. Inclusion criteria were based on our previous study [4] and as follows: (1) younger than 24 months; (2) duration of wheezing episode ≥ one month despite treatment with recommended first-line therapies of bronchodilators, inhaled corticosteroids, or systemic corticosteroids. All the included children with PW were followed up to assess the recurrence of wheezing within 24 months of recruitment. The development of wheezing was confirmed by pediatrician, mainly based on wheezing symptoms and signs. Age-matched infants underwent bronchoscopy due to bronchial granulation caused by foreign body aspiration were labeled as control group. This study is also a continuation of our previous airway microbiota study [4].

### BAL collection and sample preparation

Bronchoalveolar lavage fluid (BAL) collections were based on our previous studies [4, 20]. Bronchoscopy was transnasally performed using a flexible video-bronchoscope (XP260F, Olympus) following general anesthesia. To reduce irritation to the throat, topical throat spray with lidocaine was performed. BAL was done by instillation of sterile saline (1 ml/kg, max 20 ml). The collected BAL was centrifuged at 6000×*g* at 4 ℃ for 10 min, and the supernatant was stored at − 80 ℃ until analysis.

One hundred microliters of BAL samples were placed in an Eppendorf tube, and then added 200 µl of extract solution (acetonitrile: methanol = 1:1). The samples were vortexed for 30 s, sonicated for 10 min in ice water bath, incubated for 1 h at − 40 ℃, and then centrifuged at 12,000 rpm for 15 min at 4 ℃. The supernatant was transferred to a fresh glass vial for further ultra-high-performance liquid chromatography (UHPLC) equipped with quadrupole Orbitrap MS analysis. The quality control sample was prepared by mixing an equal aliquot of the supernatants from all of samples.

### Metabolomics analysis

Untargeted metabolomics analysis of BAL based on LC/MS was performed with a UHPLC system (Vanquish, Thermo Fisher Scientific) with a UPLC BEH Amide column (2.1 mm × 100 mm, 1.7 µm) coupled to Q Exactive HFX mass spectrometer (Orbitrap MS, Thermo). The mobile phase consisted of solution A (25 mmol/l ammonium acetate and ammonia hydroxide in water, pH = 9.75) and solution B (acetonitrile). The QE HFX mass spectrometer was used to acquire MS/MS spectra on information-dependent acquisition mode in the control of the acquisition software (Xcalibur, Thermo). In this mode, the acquisition software continuously evaluates the full scan MS spectrum [21, 22]. The parameters of ESI (electrospray ionization) were set as following: sheath gas flow rate as 30 Arb, Aux gas flow rate as 25 Arb, capillary temperature 350 ℃, full MS resolution as 60,000, MS/MS resolution as 7500, collision energy as 10/30/60 in NCE mode, spray voltage as 3600 V (positive) and − 3200 V (negative), respectively.

### Data processing and statistical analysis

The raw data of LC/MS were converted to the mzXML format using ProteoWizard software, and processed for peak detection, extraction, alignment, and integration with R language based on XCMS package (see Additional file 1). Then an in-house MS2 database (Biotree DB 2.1) was applied in metabolite identification and annotation. The cutoff for annotation was set at 0.3. The orthogonal projection to latent structures- discriminant analysis (OPLS-DA) was performed using an SIMCA software (Sartorius Stedim Data Analytics AB, V16.0.2, Umea, Sweden). The variable importance in the projection (VIP) value was calculated to summarize their contribution for each variable in the OPLS-DA model [23]. The VIP values > 1.0 were considered as significantly changed metabolites. Volcano plot was carried out to show the up-regulated or down-regulated metabolites.

All the statistical analysis and graphics were performed with R statistical software packages (R version 4.0.3). Statistical analysis was performed using descriptive statistics. The continuous variables between groups were compared by Student’s t-test or Mann–Whitney U test. Spearman rank correlations were used to demonstrate the association between various metabolites. For categorical variables, Pearson’s Chi-squared test was applied. To identify potential diagnostic biomarkers, receiver operating characteristic (ROC) curve and area under the curve (AUC) were calculated. A *P* value of less than 0.05 was considered statistically significant in all the analysis.

## Results

### Characteristics of included children

Detailed clinical characteristics between children with PW and control subjects were listed in Table 1. During this study period, 30 children with PW were enrolled and completed the follow-up. Compared with control group, children with PW had frequently a history of prematurity (6.7% vs 30%, *P* = 0.042). Of the children with PW, 17 children (56.7%) suffered from at least one episode of wheezing (recurrence) at the end of the 2-year follow-up, and 13 (43.3%) did not experience wheezing episodes (no recurrence).

**Table 1:** Characteristics of children with persistent wheezing and control children

	Persistent wheezing (n = 30)	Control (n = 30)	*P* value
Age (month)	12.63 ± 4.60	14.20 ± 3.98	0.163
Gender (F/M)	9/21	11/19	0.785
Prematurity	9 (30%)	2 (6.7%)	0.042*
Cesarean section	11 (36.7%)	13 (43.3%)	0.792
WBC counts (× 10^9^/l)	10.36 ± 3.39	9.95 ± 3.97	0.662
Neutrophils (%)	44.65 ± 21.29	38.93 ± 14.02	0.225
Hgb (g/l)	122.07 ± 9.26	121.33 ± 8.24	0.747
PLT (× 10^9^/l)	412.43 ± 142.87	366.47 ± 103.34	0.159
CRP (0–8 mg/l)	2.26 ± 4.12	2.10 ± 3.12	0.871
PCT (< 0.5 ng/ml)	0.32 ± 0.97	0.15 ± 0.14	0.635
ALT	29.03 ± 25.39	22.36 ± 10.22	0.200
AST	55.31 ± 25.77	51.21 ± 17.38	0.514

*WBC* white blood cells, *Hgb* hemoglobin, *PLT* blood platelet, *CRP* C reactive protein, *PCT* procalcitonin, *ALT* alanine aminotransferase, *AST* aspartate aminotransferase. *P < 0.05

### Metabolic profiles of BAL from children with PW

**Fig. 1:**
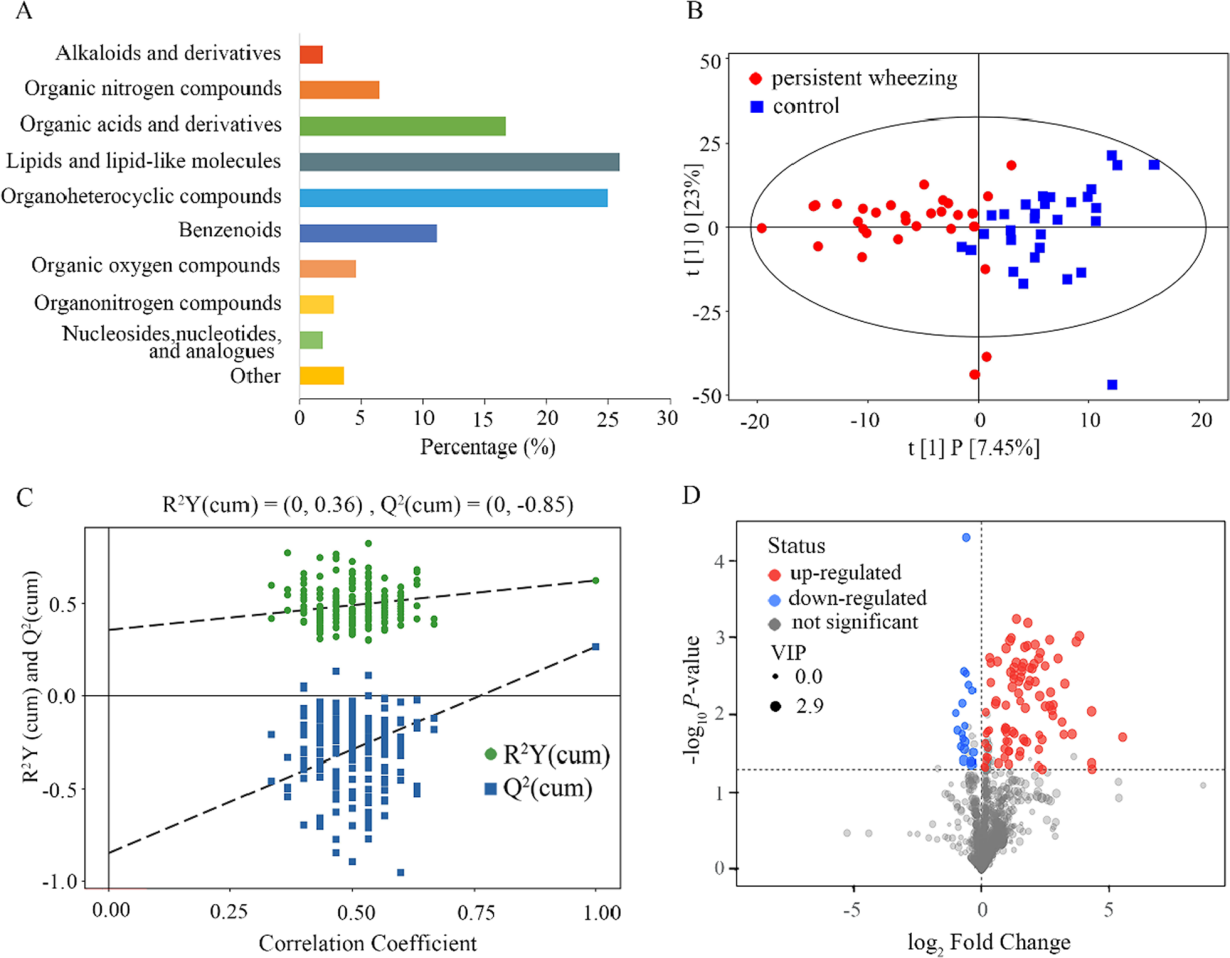
The analysis of LC/MS of BAL samples. **A** Showed the classification and proportion of metabolites. **B** Showed an OPLS-DS score plot of BAL samples between PW children and control children. **C** Showed a permutation test plot for OPLS-DA model of BAL samples. **D** Showed a volcano plot displaying the differential metabolites of PW children relative to control children. *BAL* bronchoalveolar lavage, *PW* persistent wheezing

A total of 1812 metabolite features were detected in 60 BALF samples in the untargeted LC/MS positive ion and negative ion mode, and 111 known metabolites were identified according to the reliable reference standard and annotation. These identified metabolites mainly included lipids and lipid-like molecules (25.9%), organoheterocyclic compounds (25%), organic acids and derivatives (16.7%), benzenoids (11.1%), and organic nitrogen compounds (6.5%) (Fig. 1a). As shown in OPLS-DA score plot, there was a clearer discrimination between PW children and control children (Fig. 1b). Subsequently, the permutation test of the OPLS-DA model further showed a good fit and predictive ability (Fig. 1c). The volcano plot showed the differential metabolite screening of children with PW relative to control group (Fig. 1d).

**Fig. 2:**
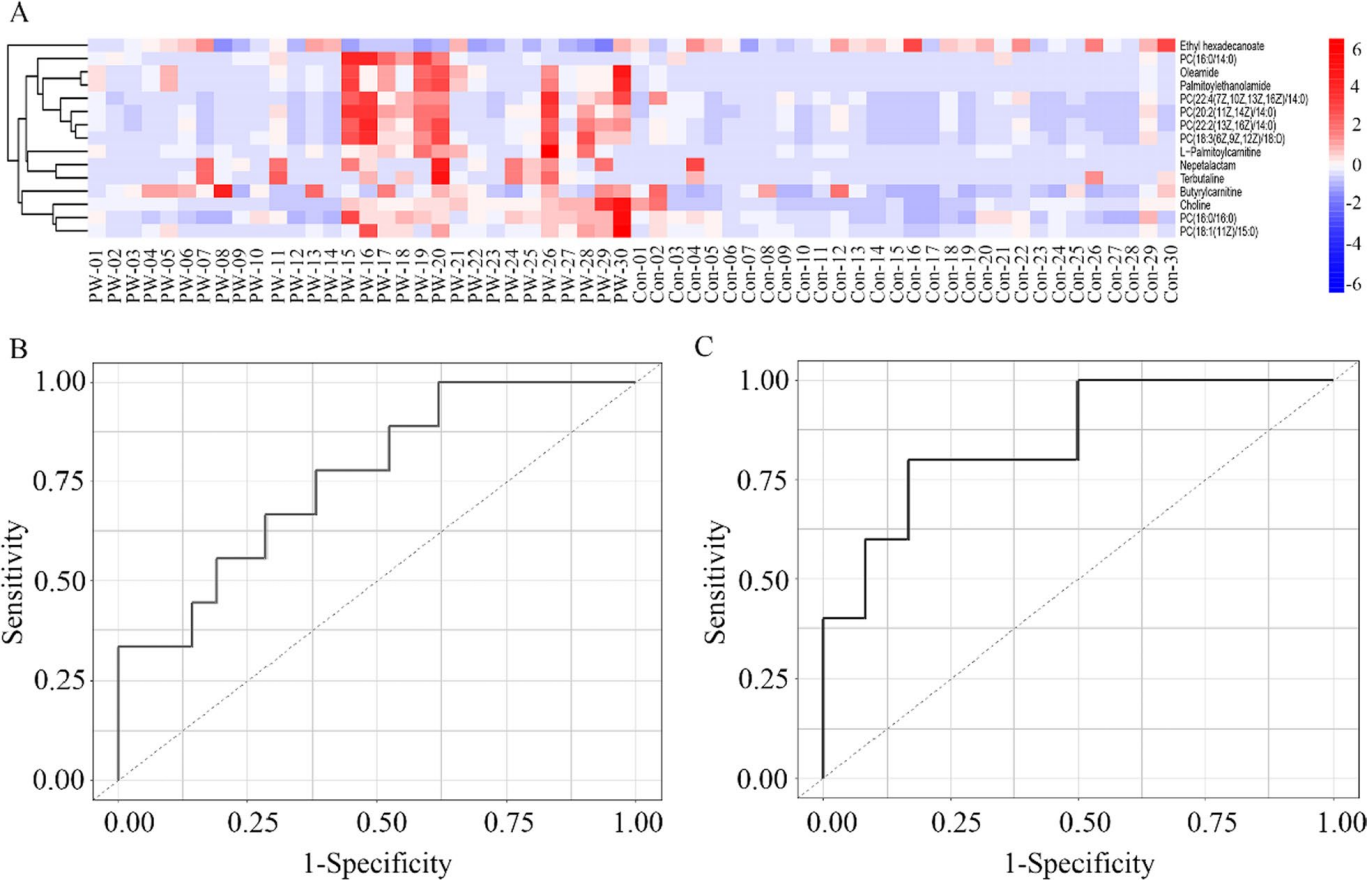
A heatmap of the identified metabolites and ROC curve analysis. **A** The heatmap showed a relative abundance of identified metabolites in each BAL sample. **B** Showed a ROC curve graph for PW children. **C** Showed a ROC curve plot for PW children with recurrence. *BAL* bronchoalveolar lavage, *PW* persistent wheezing, *ROC* receiver operating characteristic

Based on the VIP value (VIP > 1.0) and significant test (*P* < 0.05) from the OPLS-DA model, 13 metabolites (see Additional file 1: Tables S1, S2) were found to be significantly increased in children with PW compared with control group, including choline, oleamide, nepetalactam, butyrylcarnitine, l-palmitoylcarnitine, palmitoylethanolamide, and various phosphatidylcholines (PC) (Table 2). The heatmap showed the distribution of the meaningful metabolites identified in each sample (Fig. 2a).

**Table 2:** The increased thirteen metabolites in children with PW relative to control group

Metabolites	Children with PW^#^	Control group^#^	VIP	*P* value
Choline	151.396 ± 139.655	79.876 ± 67.897	1.745	0.016*
Oleamide	0.910 ± 1.272	0.069 ± 0.079	2.850	0.001**
Nepetalactam	0.465 ± 0.701	0.068 ± 0.369	2.327	0.009**
Butyrylcarnitine	0.865 ± 0.735	0.450 ± 0.524	1.911	0.015*
l-Palmitoylcarnitine	0.079 ± 0.158	0.007 ± 0.009	2.760	0.018*
PC (16:0/16:0)	41.952 ± 44.008	22.337 ± 15.756	1.705	0.027*
Palmitoylethanolamide	0.680 ± 0.941	0.047 ± 0.055	2.831	0.001**
PC (22:2(13Z, 16Z)/14:0)	4.856 ± 5.948	1.217 ± 1.148	2.432	0.002**
PC (16:0/14:0)	11.616 ± 19.959	2.398 ± 3.296	1.340	0.018*
PC (22:4(7Z, 10Z, 13Z, 16Z)/14:0)	2.238 ± 2.758	0.911 ± 0.937	1.948	0.017*
PC (18:1(11Z)/15:0)	0.555 ± 0.661	0.116 ± 0.119	1.723	0.028*
PC (18:3(6Z, 9Z, 12Z)/18:0)	0.427 ± 4.011	0.148 ± 0.671	1.945	0.030*
PC (20:2(11Z, 14Z)/14:0)	3.203 ± 25.929	0.672 ± 5.623	2.736	0.002**

^#^Data was presented with mean ± standard deviation. PC = phosphatidylcholines; PW = persistent wheezing; VIP = variable importance in the projection. *P < 0.05, **P < 0.01

### Effects of gender, birth weight, prematurity, delivery mode, wheezing history, systemic steroids use on metabolomics among children with PW

Compared with the male, the female with PW had a higher level of butyrylcarnitine (1.37 vs 0.54, P = 0.039). Relative to PW children born at term, PW children born prematurely also had a higher level of butyrylcarnitine (1.20 vs 0.54, P = 0.025, Table 3). Additionally, PW children with systemic steroids use had a higher level of l-palmitoylcarnitine compared with those without using systemic steroids (0.05 vs 0.01, P = 0.011). No significant differences were observed in other metabolites between the above subgroups. Furthermore, we find no significant differences in the metabolite levels between vaginal delivery and cesarean section groups, children with or without low birth weight, those with or without wheezing history.

**Table 3:** The comparison of metabolites between PW children with and without prematurity during the follow-up

	PW children (median, 25%, 75%)			PW with recurrence (median, 25%, 75%)		
Metabolites	Term infants (n = 21)^#^	Preterm infants (n = 9)^#^	P value	Term infants (n = 12)	Preterm infants (n = 5)	*P* value
Choline	132.77 (80.11, 176.97)	122.17 (84.08, 145.63)	0.874	135.90 (84.84, 172.05)	84.15 (68.01, 126.01)	0.292
Oleamide	0.30 (0.11, 1.10)	0.30 (0.14, 2.44)	0.769	0.23 (0.13, 1.64)	0.21 (0.05, 1.73)	0.598
Nepetalactam	0.08 (0.02, 0.92)	0.06 (0.00, 1.02)	0.946	0.31 (0.04, 1.02)	0.20 (0.002, 2.26)	0.833
Butyrylcarnitine	0.54 (0.25, 1.04)	1.20 (0.61, 1.91)	0.025*	0.47 (0.19, 0.78)	1.20 (0.68, 2.80)	0.027*
l-Palmitoylcarnitine	0.02 (0.01, 0.087)	0.02 (0.01, 0.08)	0.874	0.01 (0.005, 0.09)	0.02 (0.005, 0.11)	0.833
PC(16:0/16:0)	36.69 (16.44, 52.23)	33.73 (6.05, 56.49)	0.769	45.68 (13.06, 52.75)	7.21 (4.31, 31.01)	0.092
Palmitoylethanolamide	0.18 (0.07, 0.75)	0.31 (0.11, 1.94)	0.512	0.16 (0.05, 1.34)	0.15 (0.04, 1.48)	0.916
PC(22:2(13Z, 16Z)/14:0)	2.05 (0.90, 10.11)	1.48 (0.86, 7.51)	0.874	1.90 (0.67, 13.63)	0.92 (0.60, 9.18)	0.461
PC(16:0/14:0)	2.84 (0.58, 5.18)	1.44 (0.50, 29.06)	0.946	3.49 (0.94, 42.45)	1.44 (0.99, 20.16)	0.752
PC(22:4(7Z, 10Z, 13Z, 16Z)/14:0)	0.86 (0.62, 2.94)	0.69 (0.45, 4.14)	0.635	0.80 (0.51, 6.27)	0.48 (0.28, 2.81)	0.171
PC(18:1(11Z)/15:0)	0.17 (0.11, 0.42)	0.243 (0.12, 0.71)	0.455	0.19 (0.07, 0.38)	0.13 (0.08, 0.54)	0.673
PC(18:3(6Z,9Z,12Z)/18:0)	1.20 (0.79, 5.02)	0.91 (0.72, 3.39)	0.667	1.38 (0.54, 9.41)	0.83 (0.46, 6.06)	0461
PC(20:2(11Z, 14Z)/14:0)	9.10 (3.79, 28.02)	4.67 (2.63, 35.09)	0.635	11.34 (2.10, 72.33)	2.98 (1.68, 29.94)	0.246

^#^Term infants refer to be born at a gestational age of 37–42 completed weeks, and preterm infants were defined as any birth before 37 weeks completed weeks of gestation. PC = phosphatidylcholines; PW = persistent wheezing. *P < 0.05

### Metabolite changes between PW children with recurrence

To further understand the role of metabolomics in wheezing recurrence, we performed a two-year follow-up study. No significant differences were observed in the identified 13 metabolites between the PW children with and without recurrence. Interestingly among the PW children with recurrence, those born prematurely had a higher level of butyrylcarnitine compared with those born at term (1.20 vs 0.47, P = 0.034, Table 3). Notably, only butyrylcarnitine was found to be significantly increased in PW children born prematurely, regardless of recurrence. Among PW children, the ROC curve graph revealed that the AUC were 0.762 (*P* = 0.025, Fig. 2b) for those who born prematurely. Furthermore, for PW children with recurrence, the metabolite butyrylcarnitine possessed higher AUC values (0.85, *P* = 0.027, Fig. 2c) for those who born prematurely, indicating a better discriminatory ability.

### Rank correlation among the predominant metabolites in PW children

**Fig. 3:**
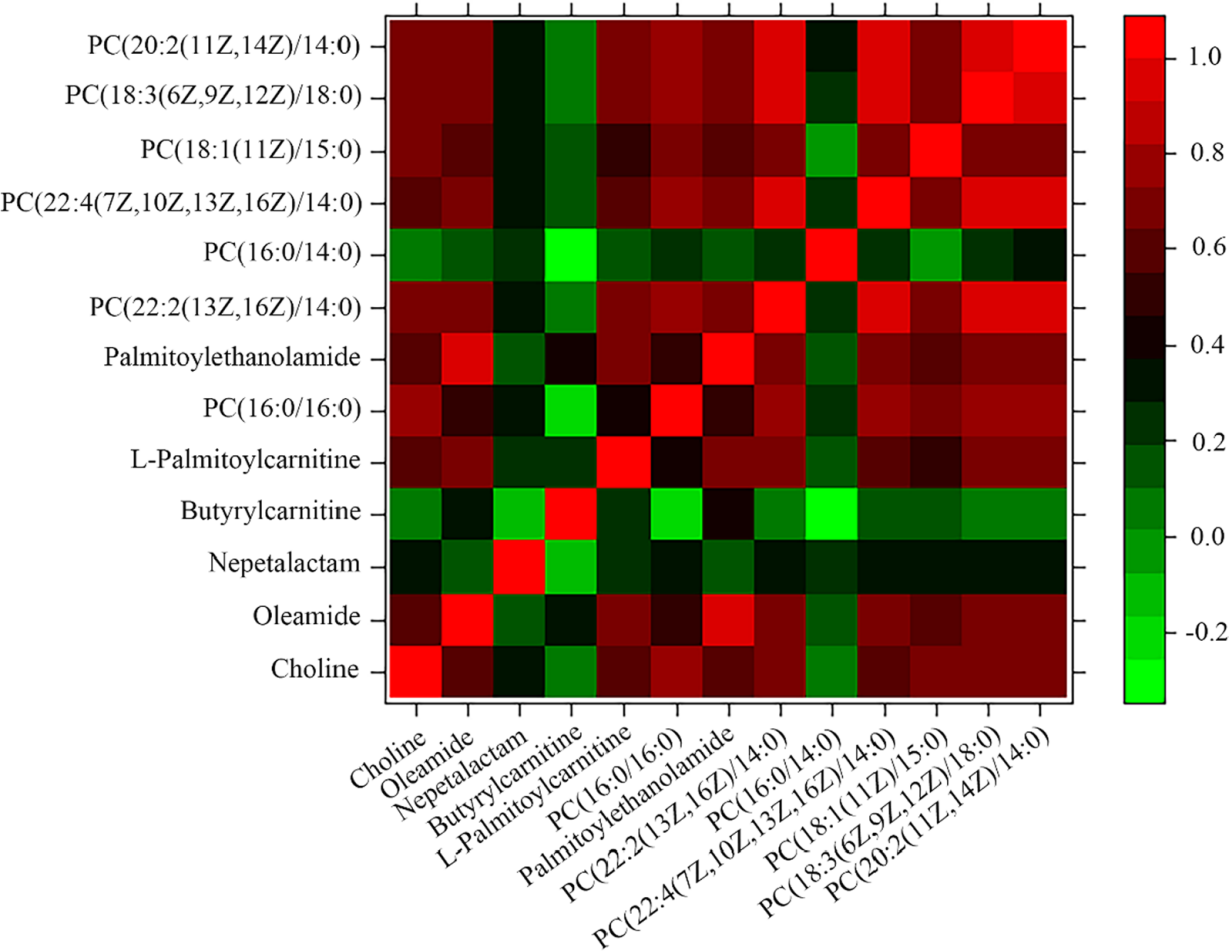
Rank correlation heatmap between various metabolites in children with persistent wheezing. The heatmap showed a correlation matrix of various metabolite abundances in children with persistent wheezing (n = 30). Red represents a positive correlation, and green indicates a negative correlation. Noted significant correlations of butyrylcarnitine with palmitoylethanolamide, l-palmitoylcarnitine with *choline, oleamide,* palmitoylethanolamide and phosphatidylcholines

Significant rank correlations between different metabolites were found among PW children (Fig. 3). The relative level of butyrylcarnitine was positively correlated with the abundance of palmitoylethanolamide (r = 0.379, *P* = 0.039). The relative abundance of l-palmitoylcarnitine was positively correlated with abundances of *choline* (r = 0.578, *P* = 0.001), *oleamide* (r = 0.719, *P* < 0.001), palmitoylethanolamide (r = 0.705, *P* < 0.001), PC(22:2(13Z, 16Z)/14:0) (r = 0.673, *P* < 0.001), PC(22:4(7Z, 10Z, 13Z, 16Z)/14:0) (r = 0.580, *P* = 0.001), PC(18:1(11Z)/15:0) (r = 0.462, *P* = 0.01),, PC(18:3(6Z, 9Z, 12Z)/18:0) (r = 0.686, *P* < 0.001), and PC(20:2(11Z, 14Z)/14:0) (r = 0.649, *P* < 0.001). In addition, we also analyzed the correlations between choline, oleamide, nepetalactam, butyrylcarnitine or l-palmitoylcarnitine and the number of wheezing recurrences. However, no significant correlations were observed (*P* > 0.05, respectively, see Additional file 1: Fig. S1).

### Pathway analysis of differential metabolites

**Fig. 4:**
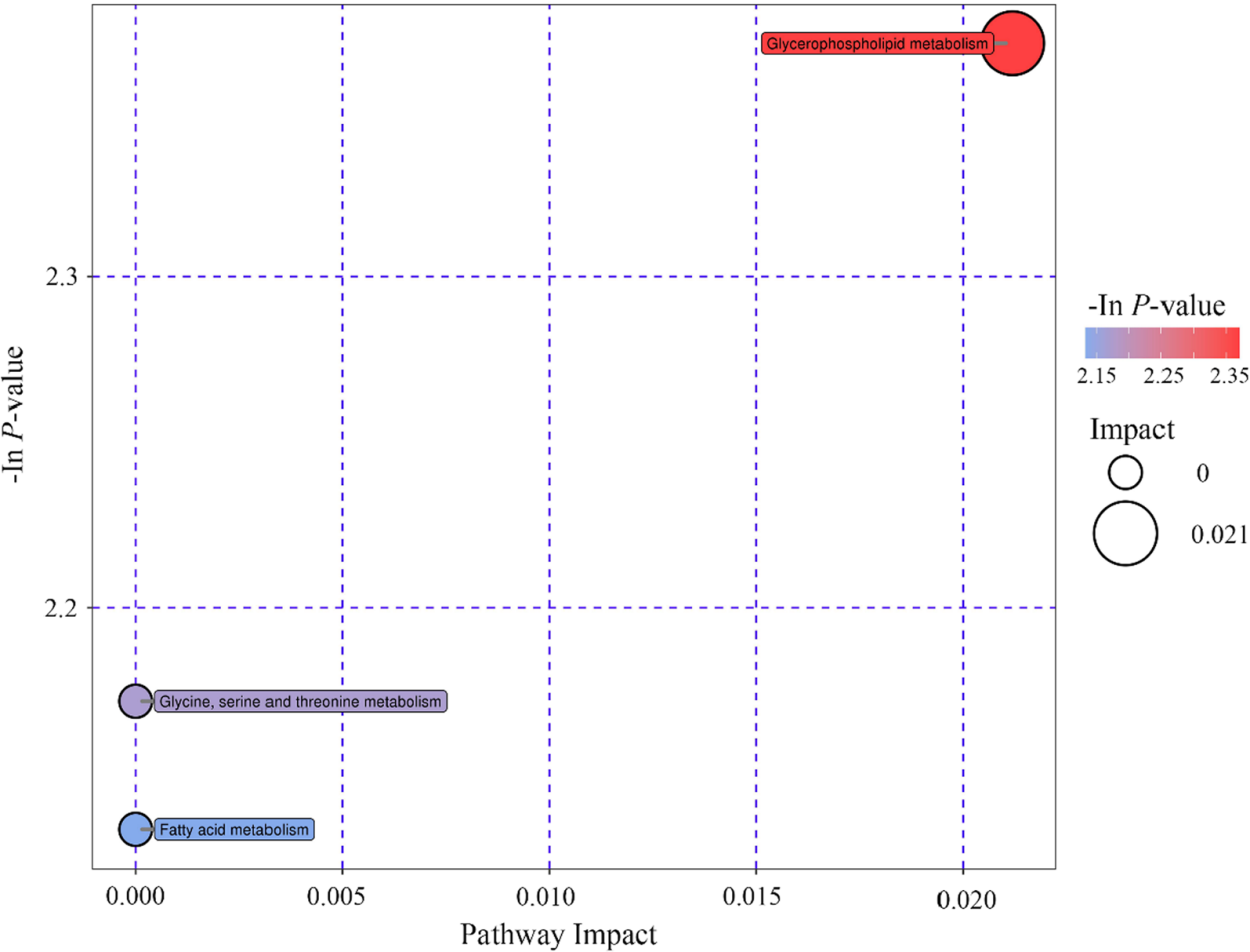
Pathway impact prediction of KEGG database in BAL samples from children with PW. *BAL* bronchoalveolar lavage, *PW* persistent wheezing

Pathway analysis for the 13 increased metabolites identified in PW children was performed by Kyoto Encyclopedia of Genes and Genomes (KEGG) Pathway database (http://www.kegg.jp/kegg/pathway.html). Three pathways including glycerophospholipid metabolism, glycine, serine and threonine metabolism, and fatty acid metabolism were revealed (Fig. 4). The glycerophospholipid metabolism pathway was the most relevant pathway with an impact factor of 0.021, indicating markedly affect persistent wheezing pathophysiologic process.

## Discussion

In the present study, younger PW children showed a distinct characterization of respiratory metabolome. The identified glycerophospholipid metabolism pathway was the most relevant pathway involving in persistent wheezing pathophysiologic process. Furthermore, for PW children with recurrence during the follow-up period, children who were born prematurely had an increased abundance of butyrylcarnitine relative to those born at term.

Untargeted metabolomics using LC/MS has been proved to be a successful strategy for finding new biomarkers for the early diagnosis and evaluation of disease [11, 24]. Analyzing samples from different compartments such as bronchoalveolar lavage were useful for the characterization of a number of chronic lung diseases, including chronic obstructive pulmonary disease, idiopathic pulmonary fibrosis and asthma [25]. Recent studies shows that metabolomics enhances the diagnostic accuracy of acute respiratory distress syndrome and asthma, and highlights the potential of metabolomics to “deep phenotype” pulmonary diseases [11]. A previous study indicated that urine sphingolipid metabolism, protein biosynthesis, and citric acid cycle were strongly associated with asthma, and hydroxybutanoic acid showed excellent discriminatory performance for distinguishing asthma from healthy controls [26]. The urinary metabolomics revealed that microbial-derived metabolites such as dimethylamine are associated with early childhood asthma, while methylnicotinamide and allantoin may participate in allergic reactions in response to allergen exposure, potentially serving as specific biomarkers for asthma [27]. Additionally, serum sphingomyelin (SM) levels were significantly decreased in asthmatic patients, particularly in asthmatics with lower blood eosinophil count, and those early-onset asthmatics (onset ages ≤ 12 years) [28]. The nuclear magnetic resonance (NMR)–based metabolomic analysis of exhaled breath condensate from children asthma demonstrated that the presence of acetylated compounds may be associated with asthma pathophysiology [14]. These studies revealed that the metabolomics of different specimens were closely related to the pathogenesis of asthma. However, the data of metabolomic pathways about infantile PW is limited, especially from BAL. Our study demonstrated that the PW children have a distinct metabolomic profiling with increased levels of choline, oleamide, nepetalactam, butyrylcarnitine, l-palmitoylcarnitine, palmitoylethanolamide, and phosphatidylcholine (PC), which could better reflect the pathogenesis of PW. Furthermore, the glycerophospholipid metabolism pathway was the most relevant pathway involving in PW pathophysiologic process.

In the present study, choline and PC were relatively the most abundant metabolites identified in BAL from children with PW. There is evidence indicating that increased serum levels of various phosphatidylcholines were associated with asthma [29]. Moreover, oxidized phosphatidylcholines may promote airway narrowing, and induce a pro-inflammatory phenotype and contraction of airway smooth muscle [30]. Additionally, choline treatment after OVA challenge via oral/intranasal routes could reverse established asthmatic conditions in mice by inhibiting airway hyperresponsiveness (AHR) and eosinophilic airway inflammation [31]. Supplemental oral choline can suppress immune inflammation and oxidative stress in asthma patients, suggesting that choline exerts anti-inflammatory effect on the airways and reduces bronchial hyper-reactivity (BHR) in asthmatics [32]. Our results further suggests that elevated choline and phosphatidylcholines levels may play an important role in the pathogenesis of persistent wheezing. On the other hand, we also found significant rank correlations between various metabolites, indicating that specific metabolomic profiling could be associated with the development or progression of infantile wheezing. Nevertheless, whether overabundance of the metabolites of BAL is the cause or consequence of persistent wheezing remains not fully understood.

Notably in this study, PW children born prematurely had a higher level of butyrylcarnitine, while PW children with using systemic steroids had a higher level of l-palmitoylcarnitine. Butyrylcarnitine is an acylcarnitine and generates during incomplete fatty acid beta-oxidation [33]. The elevated levels of serum butyrylcarnitine indicated incomplete beta-oxidation and subsequent increased reactive oxygen species (ROS) [33], and also revealed an underlying impairment of peripheral carnitine utilization and mitochondrial energy metabolism [34]. Additionally, palmitoylcarnitine is associated with mitochondrial fatty acid transport and mitochondrial metabolism [35, 36]. The palmitoylcarnitine of BAL directly inhibits the surface adsorption of pulmonary surfactant as well as its ability to reduce surface tension, representing a risk factor for lung injury [37]. These results further revealed that prematurity or systemic steroids administration showed a greater impact in airway metabolite compositions.

Most interestingly during the follow-up period, butyrylcarnitine was found to be the only elevated metabolite in PW children born prematurely, regardless of recurrence. This allowed us to speculate that there could be a clear association between airway metabolomic compositions and wheezing recurrence. Previous studies suggested that serum L-lactic acid level was significantly higher in infants with recurrent wheezing than those without; glycerophospholipid metabolism and arginine biosynthesis were the most significant changed pathways between those infants with and without subsequent recurrent wheezing [38]. The elevated urine levels of bile acid taurochenodeoxycholate-3-sulfate and fatty acid 3-hydroxytetradecanedioic acid in healthy neonates could indicate an increased risk of asthma later in life [39]. Urinary metabolomic profile can discriminate preschoolers with recurrent wheezing who will outgrow their symptoms from those who have early-onset asthma [40]. In this study, we found that among PW children with recurrence, the metabolite butyrylcarnitine possessed a better discriminatory ability for those born prematurely. This finding demonstrated that the early specific alterations in airway metabolomics are likely to indicate an increased risk of wheezing recurrence, further suggesting a close correlation between wheezing recurrence and prematurity.

Currently, there are no tools available at preschool age that can adequately predict which child will develop asthma and which child will outgrow symptoms later in life. Although some prediction rules mainly based on clinical parameters, have been developed such as the Asthma Predictive Index (API), their predictive values of these clinical indices were recently described to be low to modest [41]. Therefore, it will be promising that biomarkers identified by omics technologies predict the recurrence of wheezing along with clinical information. In addition, the present study was also a continuation of our previous study on airway microbiota of BAL. Our previous study showed that the specific airway microbiota could be strongly associated with the development and recurrence of PW [4]. Here, our present study demonstrated that the imbalance of airway metabolites could be associated with the development and recurrence of PW. These studies further suggest the important role of microenvironmental changes of alveoli and airways in the pathogenesis of PW.

The present study has some limitations that should be considered. Although metabolomics is the endpoint of the ‘‘omics cascade’’ and is the closest to phenotype, there is no single instrument platform that currently can analyze all metabolites [9]. Second, the sample size used in our study is relatively small, mainly due to the difficulty in collecting infantile specimens. Third, the influences of many other factors that might induce airway metabolomic alterations such as dietary heterogeneity, are difficult to exclude. However, we followed standard bronchoscopy procedure (fasting for 6 h before bronchoscopy) for each patient to minimize the impact of diet.

## Conclusion

The present study indicated that infantile PW showed a distinct characterization of respiratory metabolome. The increased abundance of butyrylcarnitine in the BAL of PW children suggests that the imbalance of airway metabolites could be associated with the development of wheezing. Moreover, this early alteration could also be correlated with wheezing recurrence later in life. The further development of predictive biomarkers may eventually improve an early asthma diagnosis in PW children and assist clinicians in early treatment decision-making.

## Supplementary Information

**Additional file 1: Table S1.** The original matrix of metabolomics in PW children. **Table S2.** The original matrix of metabolomics in PW children. **Figure S1.** Correlation matrix between choline, oleamide, nepetalactam, butyrylcarnitine or l-palmitoylcarnitine and the number of wheezing recurrences. No significant correlations were observed (*P* > 0.05, respectively).

## Data Availability

The datasets used and/or analyzed during the current study are available from the corresponding author on reasonable request.
