# Supplementary material for: Metabolomics of bronchoalveolar lavage in children with persistent wheezing
Source: Respir Res. 2022 Jun 19;23:161. doi: 10.1186/s12931-022-02087-6 (PMC9208141; doi:10.1186/s12931-022-02087-6)
Supplement: Supplementary file 1 — Additional file 1: Table S1. The original matrix of metabolomics in PW children. Table S2. The original matrix of metabolomics in PW children. Figure S1. Correlation matrix between choline, oleamide, nepetalactam, butyrylcarnitine or l-palmitoylcarnitine and the number of wheezing recurrences. No significant correlations were observed (P > 0.05, respectively). [file 12931_2022_2087_MOESM1_ESM.docx]

**Supplementary data**

**Methods**

***XCMS parameters***

The acquired MS data pretreatments included peak selection and grouping, retention time correction, second peak grouping, and isotopes and adducts annotation, were performed as previously described with a few modifications [1]. UHPLC-MS raw data files were converted into mzXML format using the “msconvert” program from ProteoWizard and then analyzed by the XCMS and CAMERA toolbox with R statistical software [2-4]. The CentWave algorithm in XCMS was used for peak detection. The parameter “peak-width” was set as in units of seconds, referring to the minimum and maximum peak widths for peak detection. The parameter ‘‘snthresh’’ is set as 3 for sensitive peak detection. For multiple UHPLC–MS data files, an ordered bijective interpolated warping (OBI-Warp) algorithm in XCMS was used for peak alignment. By using retention time and the m/z data pairs as the identifiers for each ion, we obtained ion intensities of each peak and generated a three-dimensional matrix containing arbitrarily assigned peak indices (retention time-m/z pairs), ion intensities (variables) and sample names (observations).

***Permutation test***

In order to avoid the overfitting of the test model and evaluate the statistical effects of the model, the permutation test was performed by randomly changing the arrangement order of the categorical variable Y and establishing the corresponding OPLS-DA model multiple times (n = 200) to obtain the R^2^ and Q^2^ values of the random model.

Table S1 The original matrix of metabolomics in PW children

| ms2nme | Choline | Oleamide | Nepetalactam | Butyrylcarnitine | L-Palmitoylcarnitine | PC_16_16 | Palmitoylethanolamide |
| --- | --- | --- | --- | --- | --- | --- | --- |
| 1 | 56.7149 | 0.060652 | 0.020599 | 0.760074802 | 0.002591 | 41.25419 | 0.056351 |
| 2 | 149.7656 | 2.702879 | 0.042449 | 0.542199001 | 0.284979 | 58.84276 | 2.270234 |
| 3 | 148.3584 | 3.150768 | 2.872135 | 1.201830396 | 0.183133 | 43.19036 | 2.638534 |
| 4 | 132.7722 | 0.095463 | 0.0612 | 0.136909881 | 0.010287 | 28.9136 | 0.076852 |
| 5 | 176.7122 | 1.387184 | 0.082645 | 0.896199788 | 0.150124 | 17.77026 | 0.930244 |
| 6 | 714.801 | 5.436938 | 0.006839 | 1.376627569 | 0.006839 | 223.8278 | 3.442228 |
| 7 | 54.5134 | 0.509174 | 1.362859 | 0.239612078 | 0.014132 | 36.68645 | 0.262453 |
| 8 | 35.77171 | 0.120501 | 0.037765 | 0.394954253 | 0.006019 | 2.562201 | 0.083546 |
| 9 | 84.14871 | 0.028553 | 1.65531 | 0.832415697 | 0.009262 | 3.7191 | 0.0228 |
| 10 | 93.56083 | 0.245742 | 0.078103 | 1.382699155 | 0.008096 | 10.96513 | 0.182358 |
| 11 | 142.893 | 1.738179 | 0.024807 | 1.748088073 | 0.113385 | 49.89038 | 1.241125 |
| 12 | 100.2303 | 0.826639 | 0.059698 | 0.458800503 | 0.050609 | 32.47026 | 0.562594 |
| 13 | 228.1455 | 0.301491 | 0.259063 | 0.339890989 | 0.065767 | 51.19061 | 0.175591 |
| 14 | 204.275 | 1.972683 | 1.111173 | 1.185991629 | 0.81243 | 50.37014 | 1.635013 |
| 15 | 217.2794 | 0.550409 | 0.004736 | 0.266706367 | 0.268373 | 62.91616 | 0.415757 |
| 16 | 103.6575 | 0.068546 | 0.002889 | 0.525330761 | 0.000475 | 18.83735 | 0.061497 |
| 17 | 84.00397 | 0.205374 | 0.202401 | 3.528512561 | 0.024499 | 4.891986 | 0.152554 |
| 18 | 122.1684 | 0.538371 | 0.390213 | 0.687999797 | 0.018817 | 33.72666 | 0.35913 |
| 19 | 95.51258 | 0.221397 | 0.000382 | 0.819964053 | 0.008337 | 19.33544 | 0.144006 |
| 20 | 177.2349 | 3.374433 | 1.320203 | 0.645473455 | 0.056892 | 138.0579 | 2.78436 |
| 21 | 128.6977 | 0.244583 | 0.061914 | 0.389934973 | 0.053884 | 63.0915 | 0.191863 |
| 22 | 81.93824 | 0.217217 | 0.358436 | 0.110594125 | 0.004994 | 25.37032 | 0.131794 |
| 23 | 156.4759 | 0.659145 | 0.482958 | 0.282446656 | 0.091579 | 49.96491 | 0.451512 |
| 24 | 527.899 | 0.627776 | 1.505991 | 1.54144414 | 0.025497 | 59.28489 | 0.537015 |
| 27 | 78.28141 | 0.085755 | 0.000136 | 1.357462705 | 0.020126 | 10.07245 | 0.049331 |
| 28 | 134.3604 | 0.054852 | 1.212031 | 0.04866421 | 0.000462 | 53.27208 | 0.034761 |
| 29 | 52.1505 | 0.304163 | 0.00037 | 2.072830102 | 0.044261 | 7.207819 | 0.314208 |
| 30 | 35.7015 | 0.029747 | 0.000348 | 0.157650802 | 0.000348 | 4.378732 | 0.016112 |
| 31 | 86.4151 | 1.371394 | 0.015849 | 1.404474736 | 0.02379 | 15.11742 | 1.132954 |
| 32 | 137.4378 | 0.162919 | 0.729003 | 0.621267742 | 0.015713 | 41.38719 | 0.043683 |

Table S2 The original matrix of metabolomics in PW children

| ms2nme | PC_22_2 | PC_16_14 | PC_22_4 | PC_18_1 | PC_18_3 | PC_20_2 |
| --- | --- | --- | --- | --- | --- | --- |
| 1 | 1.172139 | 3.91865 | 0.831752 | 0.145906 | 1.167068 | 4.480238 |
| 2 | 12.40174 | 54.84631 | 4.93775 | 0.06215 | 6.280546 | 55.88203 |
| 3 | 16.87377 | 32.89075 | 4.941636 | 0.916642 | 11.2117 | 55.20268 |
| 4 | 0.761899 | 2.841331 | 0.180575 | 0.124539 | 0.465594 | 2.055516 |
| 5 | 1.255432 | 3.252164 | 0.855264 | 0.160074 | 1.142129 | 6.727781 |
| 6 | 9.405948 | 0.006839 | 7.941277 | 3.427752 | 3.710844 | 34.04457 |
| 7 | 3.347382 | 12.28577 | 0.938492 | 0.131395 | 2.408932 | 19.29252 |
| 8 | 0.498984 | 0.510908 | 0.22721 | 0.043544 | 0.279642 | 0.797187 |
| 9 | 0.39387 | 1.305765 | 0.130924 | 0.108368 | 0.217934 | 0.656628 |
| 10 | 1.177762 | 1.870658 | 0.688257 | 0.167325 | 0.717753 | 7.350866 |
| 11 | 5.620399 | 39.07835 | 1.816633 | 0.452612 | 3.062924 | 35.78658 |
| 12 | 2.049379 | 3.657644 | 1.454936 | 0.286338 | 0.9854 | 8.874413 |
| 13 | 1.702052 | 2.31679 | 0.490876 | 0.406694 | 1.440207 | 9.100682 |
| 14 | 14.48999 | 0.284541 | 9.623115 | 0.617892 | 11.60828 | 77.80843 |
| 15 | 11.23232 | 0.126343 | 2.540757 | 0.826666 | 10.36391 | 36.75365 |
| 16 | 0.804053 | 7.433064 | 0.424962 | 0.129919 | 0.705609 | 4.671291 |
| 17 | 0.915102 | 0.681017 | 0.483819 | 0.057741 | 0.906572 | 2.695247 |
| 18 | 0.922029 | 0.317109 | 0.512751 | 0.238863 | 0.730095 | 2.560116 |
| 19 | 0.548106 | 4.138296 | 0.568239 | 0.097647 | 0.476718 | 1.763424 |
| 20 | 14.04912 | 67.80757 | 6.715755 | 0.236689 | 10.46301 | 85.54967 |
| 21 | 3.584873 | 25.23854 | 3.330483 | 0.503839 | 3.013087 | 34.39111 |
| 22 | 1.039608 | 5.25607 | 0.696818 | 0.135518 | 0.867966 | 3.099951 |
| 23 | 22.27957 | 66.33056 | 8.84622 | 1.629375 | 13.69259 | 83.76034 |
| 24 | 8.997647 | 0.00395 | 3.333523 | 0.689158 | 3.756686 | 14.01354 |
| 27 | 3.317597 | 0.528728 | 0.719903 | 0.078729 | 1.197432 | 9.995167 |
| 28 | 2.088259 | 2.840471 | 0.909373 | 0.287554 | 1.321781 | 13.58108 |
| 29 | 1.480602 | 1.442186 | 0.688083 | 0.164235 | 0.830031 | 2.979279 |
| 30 | 0.252091 | 0.624959 | 0.222908 | 0.043272 | 0.191907 | 0.62392 |
| 31 | 0.661433 | 1.545875 | 0.665355 | 0.43962 | 0.911053 | 7.137237 |
| 32 | 2.34212 | 5.108935 | 1.40844 | 0.212559 | 1.961217 | 14.83808 |

Figure S1 Correlation matrix between choline, oleamide, nepetalactam, butyrylcarnitine or L-palmitoylcarnitine and the number of wheezing recurrences. No significant correlations were observed (*P* > 0.05, respectively).


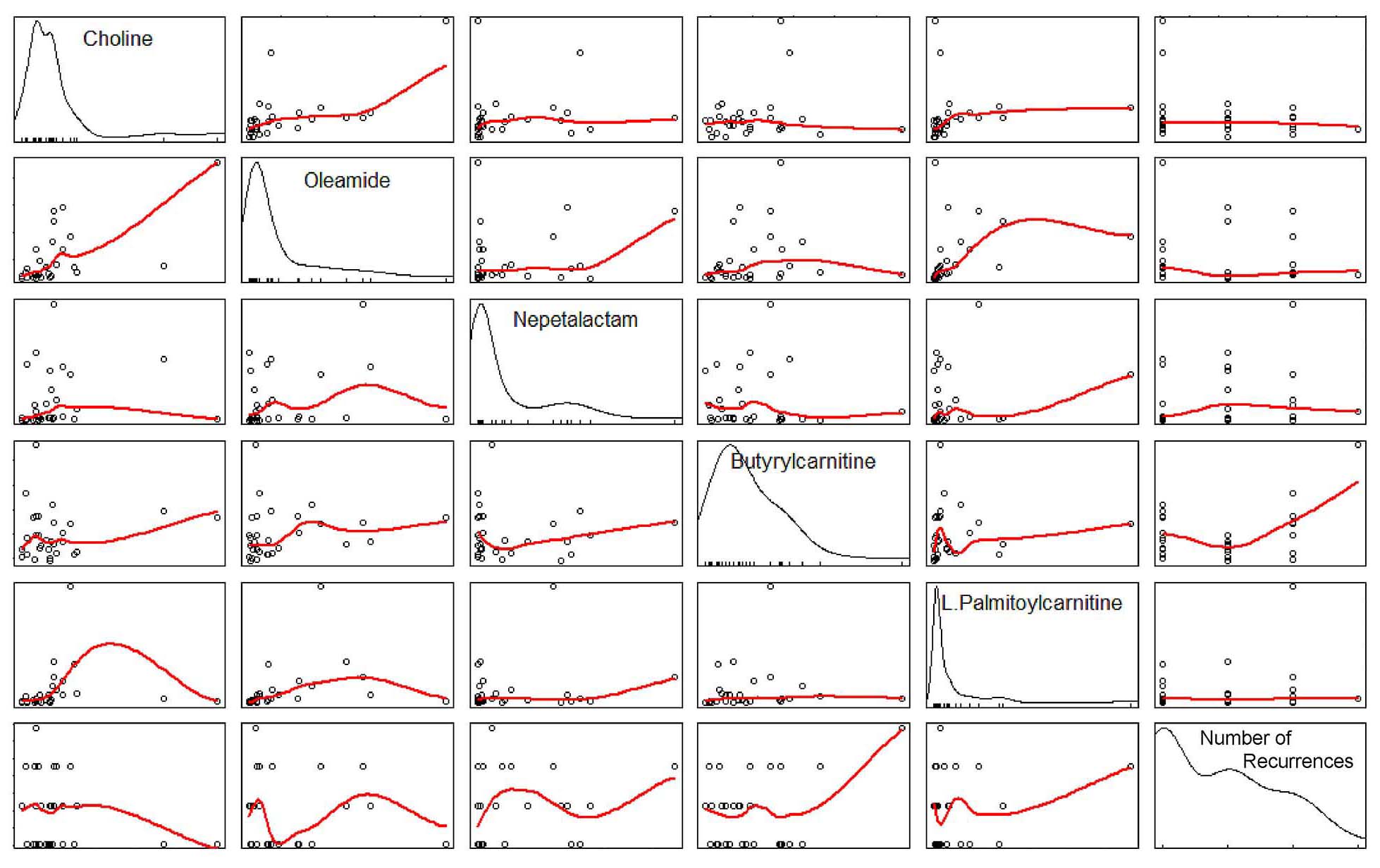


**References**

1. Smith CA, Want EJ, O'Maille G, Abagyan R, Siuzdak G: XCMS: processing mass spectrometry data for metabolite profiling using nonlinear peak alignment, matching, and identification. Anal Chem. 2006; 78 (3):779-787.

2. Prince JT, Marcotte EM: Chromatographic alignment of ESI-LC-MS proteomics data sets by ordered bijective interpolated warping. Anal Chem. 2006; 78 (17):6140-6152.

3. Tautenhahn R, Bottcher C, Neumann S: Highly sensitive feature detection for high resolution LC/MS. BMC Bioinformatics. 2008; 9 504.

4. Liu R, Hong J, Xu X, Feng Q, Zhang D, Gu Y, Shi J, Zhao S, Liu W, Wang X, et al: Gut microbiome and serum metabolome alterations in obesity and after weight-loss intervention. Nat Med. 2017; 23 (7):859-868.
